# Research on Pantograph Defect Classification Based on Vibration Signals

**DOI:** 10.3390/s24237741

**Published:** 2024-12-03

**Authors:** Vytautas Gargasas, Kęstas Rimkus, Mindaugas Alekna, Andrius Knyš, Mindaugas Žilys, Algimantas Valinevičius

**Affiliations:** Faculty of Electrical and Electronics Engineering, Kaunas University of Technology, LT-51367 Kaunas, Lithuania; kestas.rimkus@ktu.lt (K.R.); abrikosas1@gmail.com (M.A.); andrius.knys@ktu.lt (A.K.); mindaugas.zilys@ktu.lt (M.Ž.); algimantas.valinevicius@ktu.lt (A.V.)

**Keywords:** train pantograph, classification features, Mel spectrograms, convolutional neural networks

## Abstract

Pantograph-based electrical current transmission systems are used in electric traction vehicles. The contact surface between the pantograph and the catenary wire experiences mechanical and thermal effects during the train’s movement. Typically, this contact surface on the pantograph is covered by a segmented carbon or copper rod, attached to an aluminum base. Railways implement organizational measures for pantograph condition monitoring, based on scheduled inspections. Constitutionally, the option to replace contact elements or individual segments of the pantograph exists if wear is detected. Many scientific publications describe ideas for pantograph visualization and automated condition monitoring. These ideas are based on analyzing mechanical vibrations generated by the pantograph, acoustic vibration signal analysis, 3D geometric data of the pantograph surface captured by laser scanning, and combinations of several methods. However, in these publications, mechanical vibration analysis is limited to signal shape and spectral analysis. The possibility of treating the vibration signal as a random process using statistical methods has not been utilized. This study describes the possibility of evaluating classified mechanical pantograph vibrations using the signal’s autocorrelation transformation. A laboratory experiment confirmed the proposed method for evaluating informative signal classification features. The proposed method can distinguish between signals generated by a defective pantograph surface and identify different types of defects.

## 1. Introduction

Analyzing the evolution of pantograph diagnostics and visualization ideas in electric trains reveals a trend of changes in solutions and technologies used. In publications [1,2,3,4,5,6,7], pantograph diagnostics involved analyzing acoustic or mechanical vibration signals. Later [8,9], methods based on the analysis of captured 2D pantograph images (photos) were proposed, and later publications suggested supplementing visualization and condition evaluation systems with 3D laser scanning analysis [9,10]. This evolution in technical tools was driven by advances in hardware performance: the computer analysis of 3D image data would have taken an impractically long time, but now results can be obtained faster, allowing timely responses to detected damage, such as unscheduled pantograph inspections or even immediately halting a train if a defect is deemed dangerous. Later publications, such as one in 2014 [11], analyze the curve of the pantograph surface shape captured by a 3D laser scanner, using various mathematical transformations to detect informative features indicating the pantograph’s condition. This development in diagnostic methods suggests revisiting earlier attempts to analyze mechanical–acoustic vibrations. While vibrations do not directly correspond to the 3D shape of the pantograph’s surface, diagnostic signals may still provide features indicating pantograph condition. Notably, the ideas from 2005 to 2007 only involved evaluating the vibration signal’s shape, with spectrum analysis as the sole transformation. However, a 2017 study [3] used other mathematical transformations, including mutual correlation and autocorrelation functions. The autocorrelation function is known to be effective in classifying diagnostic signals, especially when the signal is non-stationary and has a complex shape. This study aims to determine whether the analysis of pantograph-generated vibrations can identify classification features with sufficient accuracy to detect not only the presence of defects but also their nature. 

In a 2024 publication [12], an in-depth analysis of high-speed train pantograph vibrations was presented. Although it was not aimed at solving diagnostic issues, the experimental data curves may contain signals potentially useful for condition diagnostics. The study by Guangning Wu [13] discussed pantograph system diagnostics in terms of contact wire condition, temperature monitoring, visual surveillance, 3D modeling tools, and even electromagnetic diagnostics. This paper also mentions the idea of using acoustic vibrations for diagnostics, which can be compared to the principles of mechanical vibration diagnostics. However, these studies are still limited to spectrum analysis, possibly due to this limitation, yielding insufficient results. It is crucial to investigate whether additional signal transformations could yield more accurate diagnostic features. This paper aims to analyze the mechanical vibrations generated by the pantograph to derive features suitable for diagnostics. The innovation lies in the fact that only the vibration signal’s waveform or spectrum analysis is used for diagnostics. This type of vibration signal analysis has not been conducted before, and the obtained results indicate that it is possible to enhance the effectiveness of existing diagnostic systems, aiming to avoid the use of complex and expensive video or laser diagnostic systems.

## 2. Materials and Methods

It is known that many objects emit signals during operation that could be applied to diagnostics. These could be mechanical or acoustic vibrations, temperature changes, or other parameter fluctuations that depend on the object’s condition. Sometimes, objects do not emit such signals during regular operation, but they can be induced using specific stimuli. The pantograph of an electric train has both mechanical and electrical contact with the catenary wire. During train movement, mechanical effects and sparking cause mechanical vibrations. Additionally, the contact point on the pantograph surface changes position cyclically, moving back and forth along the pantograph’s length. This movement generates vibrations and acoustic noise in the surrounding environment. However, acoustic signal detection is complicated by other noises generated by the moving train. Distinguishing diagnostic signals from pantograph-specific ones acoustically is challenging. This study focuses on analyzing mechanical vibrations captured by a piezoelectric sensor. Properly attached to the pantograph or supporting structures, the piezoelectric sensor can detect pantograph-induced vibrations with a stronger signal than ambient mechanical noise. Although the sensor attachment method is not new, studies on captured signals have been limited to signal shape and spectral analysis. A laboratory setup was developed for this study to record pantograph-induced vibrations in a controlled environment. The setup included a stretched catenary wire segment between stands, with a piezoelectric sensor attached to the wire’s support structures. 

**Figure 1:**
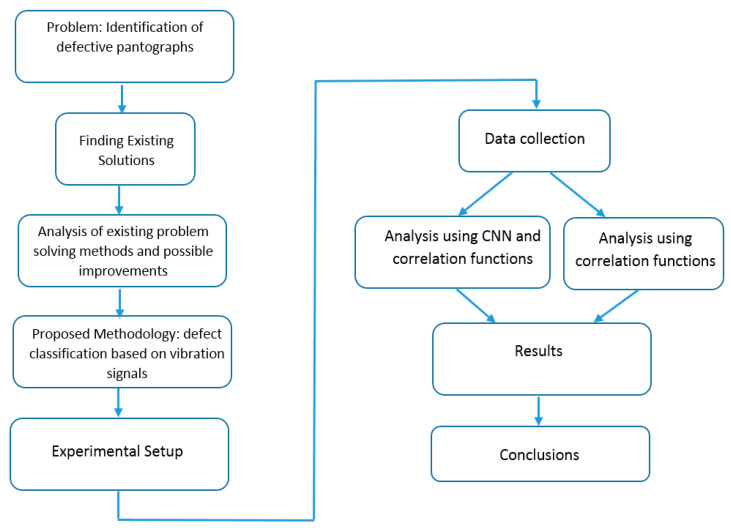
Flowchart of research ideas and experimental methods.

A flowchart of research ideas and experimental methods of this article is presented in Figure 1.

**Figure 2:**
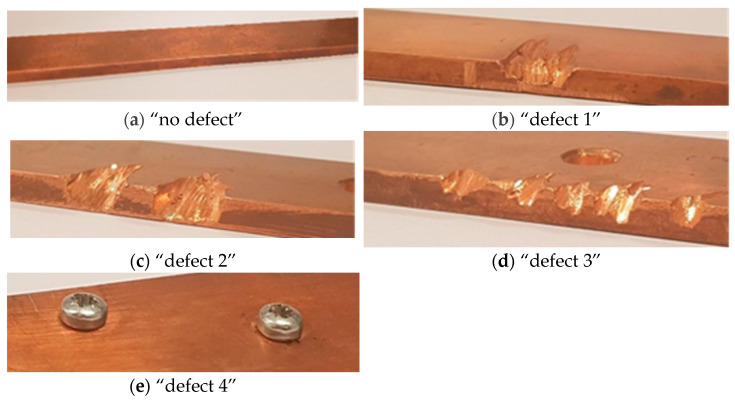
Simulated surface shapes for (**a**) “no defect”; (**b**) “defect 1”; (**c**) “defect 2”; (**d**) “defect 3”; (**e**) “defect 4”.

In creating the experimental conditions, the train’s speed was selected at which the vibration signals were recorded. According to the catenary system design, the current transmission wire is suspended at a small angle relative to the track axis, and this angle changes at each support structure that contains wire suspension elements. Therefore, as the train moves from one support structure to the next, the contact point of the wire on the pantograph surface sequentially shifts from one edge of the pantograph’s contact surface to the other, and then, after passing the support, the contact point moves back in the opposite direction. During the experiment, the process of contact point movement was simulated, and mechanical vibrations caused by irregularities in the pantograph’s surface were recorded during this movement. To determine the contact point’s movement speed during the experiment, it is necessary to select the train’s speed and evaluate the relationship between the pantograph’s contact point with the wire and the train’s speed. It is understood that the induced vibrations can vary depending on the train’s speed; therefore, the experiment was limited to a train speed corresponding to a situation where the train is traveling between stations at cruising speed. In real situations, this speed may change depending on traffic conditions and the train schedule. For this experiment, the assumption was made that the mechanical vibrations being examined are from a train traveling at a speed of 24 km/h as the average speed of the train approaching a station checkpoint lies between 20 and 40 km/h. The length of the pantograph’s contact surface was noted in reference materials as 1120 mm. The distance between supports was assumed to be 60 m. Thus, when the train has traveled 60 m, the contact point can move up to 1.12 m. In real conditions, the distances between supports may vary slightly, and the contact wire may not touch the pantograph along the entire surface length but only part of it. Nevertheless, for the experiment, the assumption was made that these parameters remain consistent during the train’s movement. Therefore, with the train moving at a speed of 24 km/h, the pantograph’s contact point movement speed was 0.448 km/h or 0.124 m/s. The simulated train speed was within the range of 20 to 40 km/h during the laboratory test. Contact surfaces simulating a healthy surface and various levels and types of mechanical damage, from indentations to protrusions, were used during the experiment, and images of these surfaces are shown in Figure 2.

The most common pantograph surface deformation defects described in the literature have been replicated in the segments of the pantograph’s contact surface. The literature analysis revealed that there are not many types of pantograph defects. Several defect types are mentioned in the literature, which are most commonly encountered when operating electric traction vehicles. The vast majority of defects manifest as wear of the pantograph material on its contact surface. Most often, these are one or several nearby indentations on the pantograph surface (defects 1, 2, 3). Another defect mentioned in the literature is the formation of a protrusion on the copper pantograph surface, which occurs due to the thermodynamic properties of the pantograph surface—defect 4. To simulate a defective pantograph, imitation copper plates were created, replicating the known defective pantograph surface shapes. Automated object diagnostic systems are typically based on the principle of signal feature classification. The creation of the system starts with a set of signal features from healthy (defect-free) objects. Later, the features of the tested signal are compared with the features of the healthy objects’ signals. If the features do not match, the object being tested is classified as defective. If it is desired that the system not only distinguish a defective object from a healthy one but also identify the type of defect (or even the level of risk it poses), classifier training is performed during the system’s setup. In total, 128 pantograph-generated mechanical vibration signals were collected: 22 signals from a healthy pantograph, 23 from defect type 1, 23 from defect type 2, 25 from defect type 3, and 25 from defect type 4. To register the vibrations, industrial vibration sensor RS PRO, 10 kHz, ±1 dB, was used [14]. 

**Figure 3:**
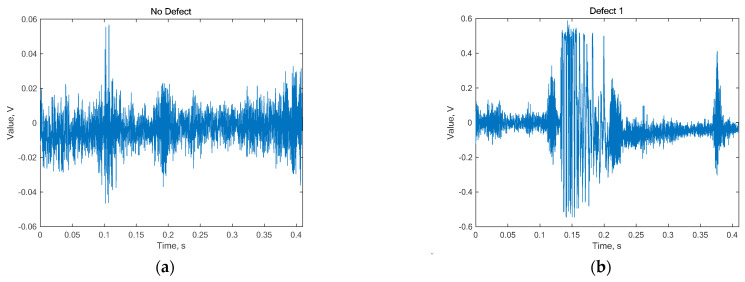
Vibrations generated by a healthy pantograph (**a**) “no defect” and (**b**) “defect 1”.

The frequency of the registered pantograph vibration signal was 0 to 10 kHz, with a signal level of 32,767 corresponding to 1 V, or vice versa, where 1 digit corresponds to 3.05181 × 10^−5^ V. One example of such a healthy signal (a) and the first defect type (b) is shown in Figure 3. 

**Figure 4:**
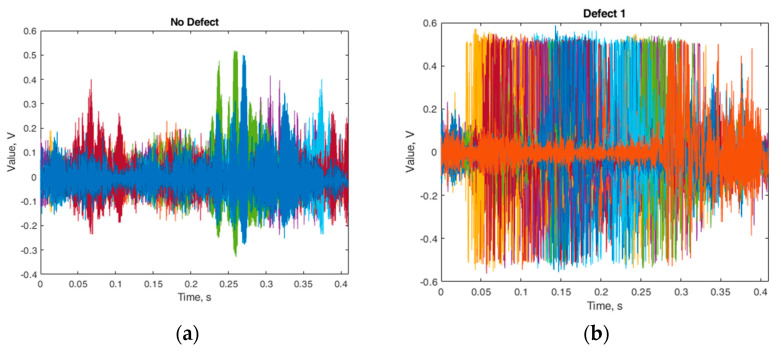
All vibration signals are generated and plotted in the same coordinates by a healthy pantograph (**a**) “no defect” and (**b**) “defect 1”.

Although these individual signals are quite different, as can be seen from the graphical representation of all recorded signals in Figure 4, the difference between the healthy contact surface (a) and defect 1 (b) was no longer as apparent, thus requiring more detailed analysis to distinguish such signals. Each color in Figure 4 represents a different pantograph signal for the same defect. The purpose of using different colors was to distinguish each measurement as separate; if all measurements were represented in a single color, they would appear as one unified measurement. It was necessary to display all measurements on a single graph to highlight the difference compared to the graph in Figure 2b, showing that the peak from “Defect 1” could occur at any moment.

## 3. Results

Various classification algorithms exist, and the required number of features to ensure reliable classifier performance was determined. This study tested a CNN classifier and proposed a method for extracting features from pantograph-induced vibrations. These features must be detectable in the vibration signal and quantified.

### 3.1. Statistical Signal Analysis

**Figure 5:**
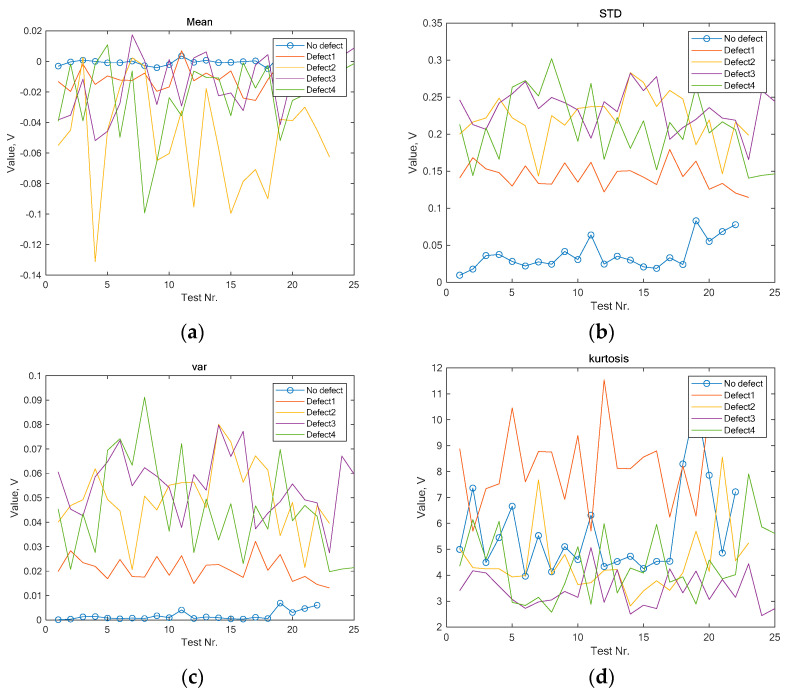
Statistical evaluations of the vibrations generated by the pantograph signals: (**a**) mean; (**b**) std; (**c**) var; (**d**) kurtosis.

Based on the signal forms in Figure 3, it is difficult to directly draw conclusions about any drive characteristics. The most appropriate way to analyze such signals is to begin with basic statistical parameter evaluations, such as mean, standard deviation, variance, and kurtosis, with the results presented in Figure 5.

As can be seen from the results in Figure 4, calculating the mean standard deviation and variance allows one to easily distinguish the signal from a healthy contact surface from that of defective signals. However, distinguishing between different defects using this information alone is impossible, as their signals overlap. Calculating the mean value of the signals or the kurtosis index also does not clearly differentiate even the vibration signal generated by a healthy surface. Additional studies using other statistical metrics, such as skewness, RMS, and the peak-magnitude-to-RMS ratio, were also conducted, but the results were similar. 

### 3.2. Mel Spectrograms and CNN Signal Analysis

**Figure 6:**
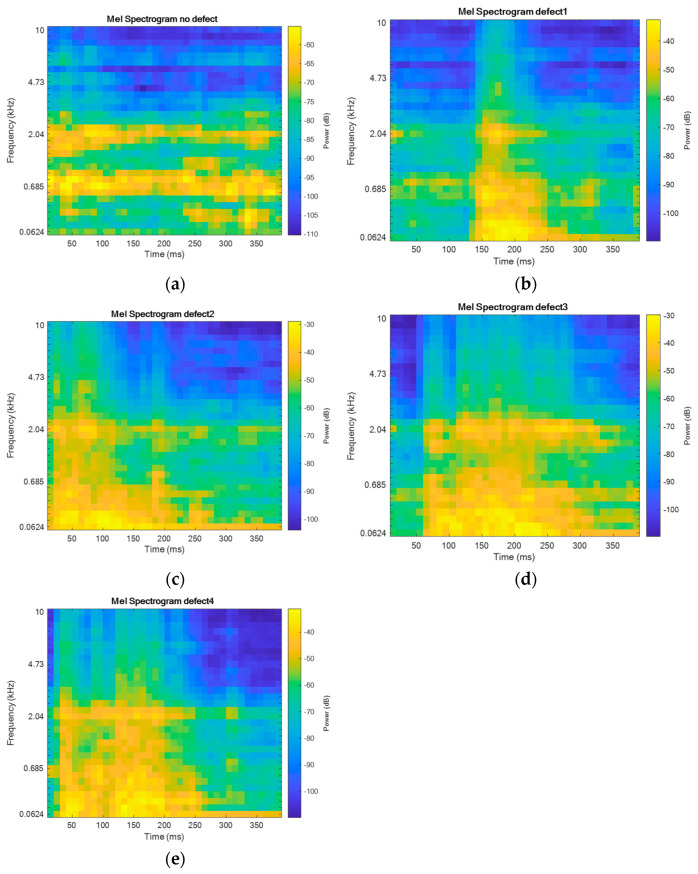
Mel spectrograms of vibrations generated by the pantograph signals for (**a**) “no defect”; (**b**) “defect 1”; (**c**) “defect 2”; (**d**) “defect 3”; (**e**) “defect 4”.

As shown by the literature analysis, Mel spectrograms and CNN (convolutional neural networks) models trained on them are often used for sound signal analysis. Therefore, for further signal analysis, it was decided to use Mel spectrograms, as they allow the identification of frequency components that may indicate defects not visible in the time domain. The Mel scale better reflects small frequency changes that can be important for identifying wear or other structural defects. Additionally, the spectrogram shows how the signal frequency changes over time, allowing for more efficient classification of defects based on their size and nature. An example of a Mel spectrogram for each signal type is provided in Figure 6.

**Figure 7:**
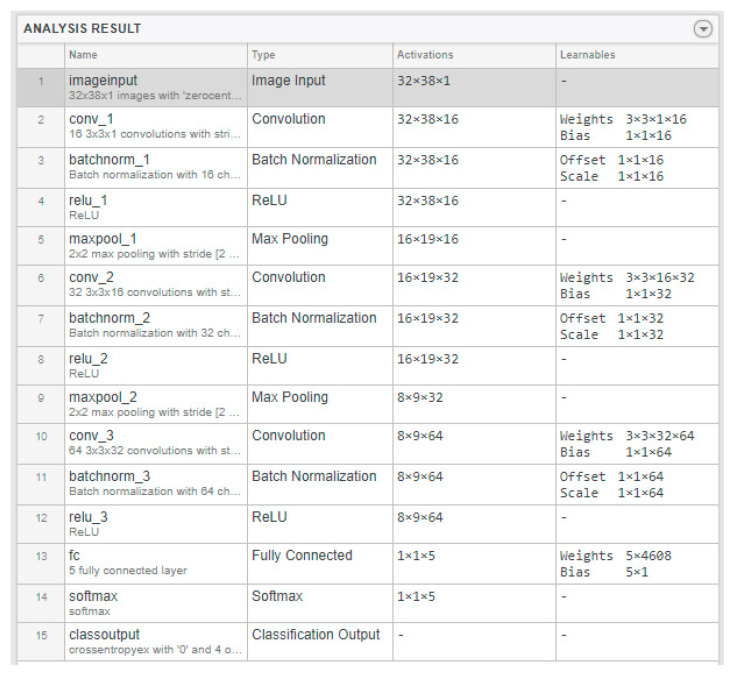
Structure of the CNN used for the classification of Mel spectrograms of vibrations generated by the pantograph signals.

Mel spectrograms as images were used to train and test CNN. The classification of the signals’ Mel spectrograms was carried out using a CNN with a ReLU activation function. The entire structure of the CNN used for classification is presented in Figure 7.

**Figure 8:**
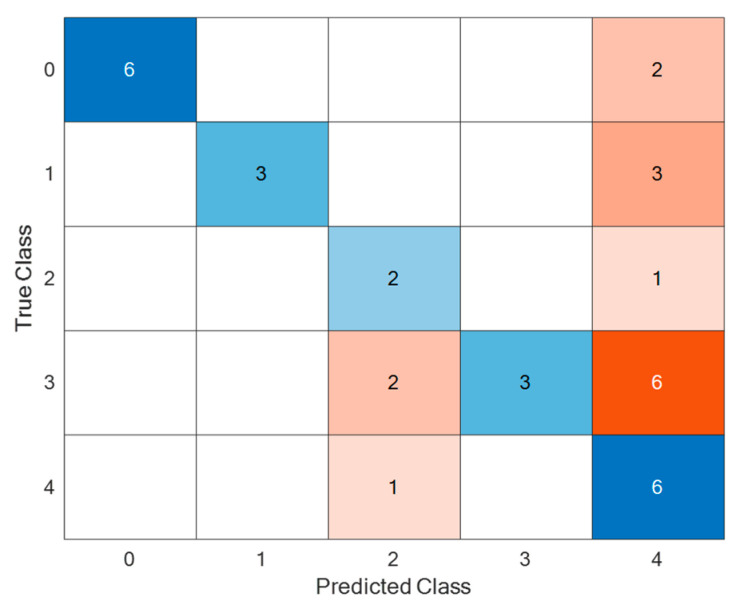
Classification of Mel spectrograms of vibrations generated by the pantograph signals using a trained CNN on test data.

The signals from each class were divided with a ratio of 70% (for training) and 30% (for testing). With this dataset, after 20 epochs, the CNN training stopped because a stable value was reached. The confusion matrix of the obtained results is presented in Figure 8.

As seen in Figure 8, the healthy pantograph signal is classified with an accuracy of 85.7%, while the overall average classification accuracy for all classes is 68.6%. Blue color represents correct classification results, red – incorrect classification results. The obtained results essentially reflect those achieved using basic statistical evaluations (standard deviation and variance), meaning that the healthy signal is classified most accurately, while defects of different levels are mixed together. Considering that the successful training of CNN methods is typically based on large datasets, and in our case, training with approximately 15 samples yielded an average recognition accuracy of 68.6%, it is likely that accumulating a larger dataset could significantly improve this classification accuracy. 

### 3.3. Stochastic Signal Analysis

Furthermore, stochastic signal analysis tools were employed to analyze these signals, along with additional mathematical functions that allow the signal to be transformed into a more deterministic form. The most common of these transformations is the Fourier transform:

(1) $$f(x)=\int_{-\infty }^{\infty }F\left(k\right)e^{2i\pi kx}dx,$$

also known as spectral signal analysis.

The signal spectrum would reveal the frequency characteristics of the signal, and the classifier could operate by comparing the predominant harmonics of the signal. Additionally, there are methods for signal transformation based on other mathematical functions, such as the correlation function:

(2) $$R_{f}\left(\tau \right)=\int_{-\infty }^{\infty }f\left(v+\tau \right)f\left(v\right)dv,$$

The autocorrelation function, the signal cepstrum:

(3) $$\hat{x}=\frac{1}{2\pi }\int_{-\infty }^{\infty }log\left[X\left(e^{j\omega }\right)\right]e^{j\omega n}d\omega ,$$

and more.

In order to identify the characteristic features that enable an accurate evaluation of the drive’s vibration signal, the aforementioned transformations were performed using the MATLAB software package.

To find the transformation method, the vibration signals generated by both the healthy and defective pantographs were analyzed. The goal was to obtain an identity function through transformation that changes as consistently as possible and for the values of the identity functions at those coordinate points to serve as classification features where the influence of different defects on the transformed signal’s shape is most distinct.

**Figure 9:**
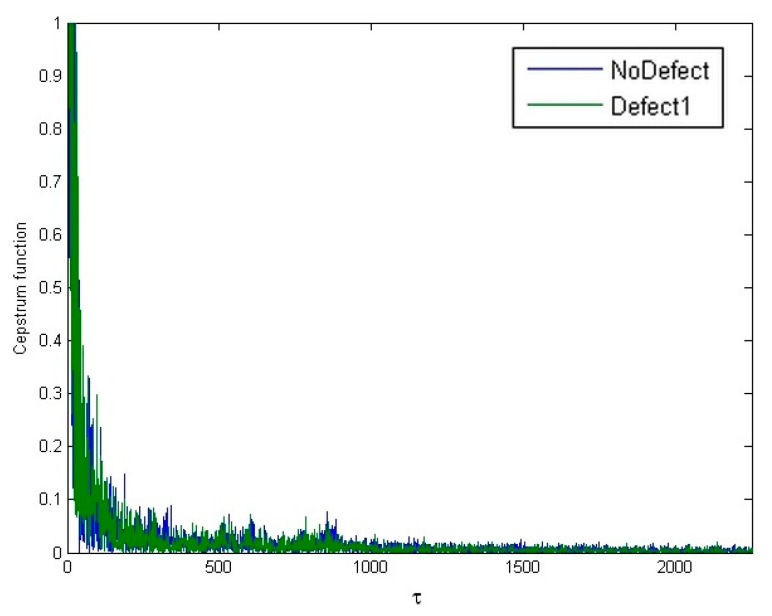
Cepstrum functions of “no defect” and “defect 1” signals.

**Figure 10:**
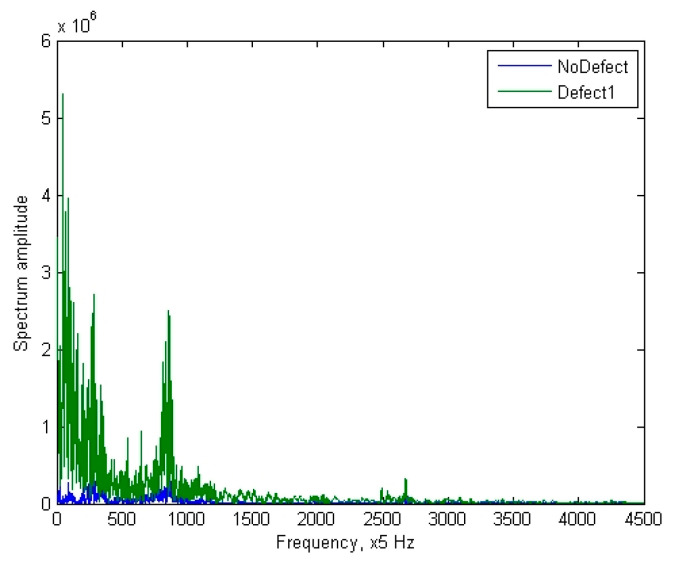
Spectrum of the “no defect” and “defect 1” signal.

**Figure 11:**
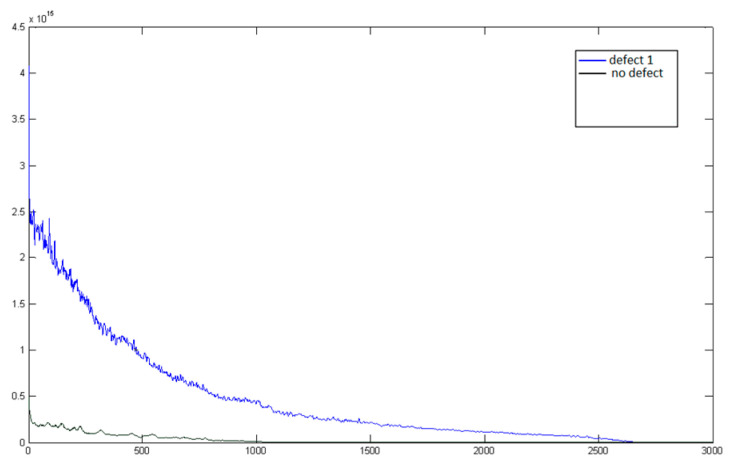
Autocorrelation functions of “no defect” and “defect 1” signal spectra.

From the transformations of the analyzed signals, it was observed that neither the autocorrelation function of the signal nor the signal cepstrum, represented in Figure 9 yields the desired result: the signal remains difficult to define (non-deterministic). In such a signal form, it is still challenging to begin searching for defining features of the signal, making it difficult to evaluate them quantitatively. The signal spectrum, represented in Figure 10, while also having very unstable values in each frequency range, nonetheless has several extrema in its form, and it seems that the frequency or amplitude of these harmonics could already be assessed as a feature reflecting the condition of the pantograph. However, this is still not a fully suitable signal form for feature evaluation, and therefore, combined signal processing methods were investigated. Often, when the direct cepstrum or autocorrelation of the signal is difficult to define, a combinational transformation is used: first, the signal spectrum is obtained, and only then is the spectrum transformation performed. The autocorrelation function of the signal spectrum has a clearly defined form and changes relatively consistently, as represented in Figure 11 (axes values are dimensionless).

After determining the appropriate signal transformation method, several samples were examined from each of the four pantograph contact surface models exhibiting different defects.

By plotting the transformations of the vibration signals caused by the healthy pantograph and the four different defects using the spectrum autocorrelation method, it is evident that the identity functions differ from one another, and there are specific coordinate points where the values of the identity functions for the different defects vary.

**Figure 12:**
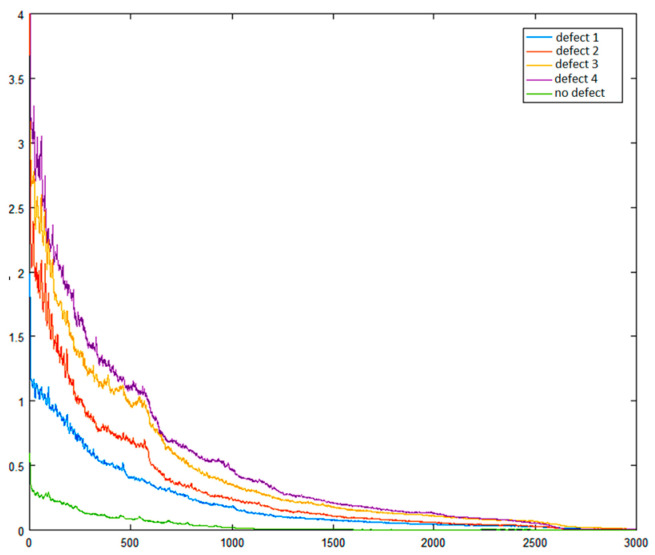
Identity functions of the signals.

The results obtained reveal both the advantages and disadvantages of the method represented in Figure 12 (axes values are dimensionless). It is clear that the features of the vibration signal from a healthy pantograph have the lowest numerical values, making it suitable to classify the object as either defective or healthy. However, the features of individual defect classes, while differing to some extent, are still very close to each other.

**Figure 13:**
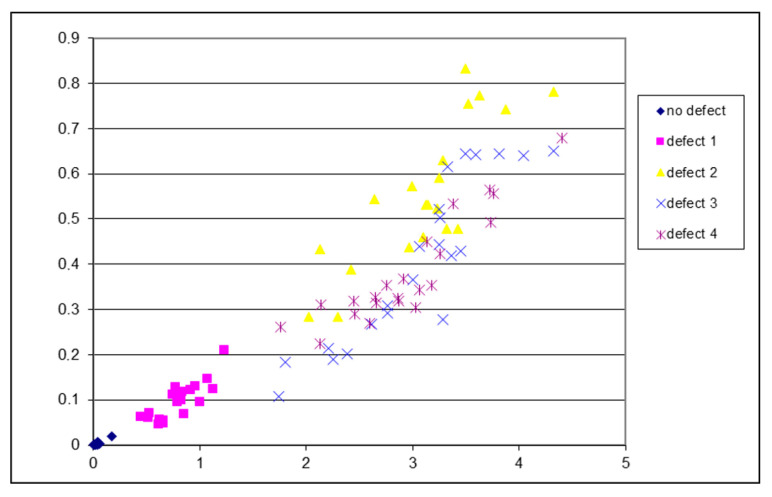
Distribution of signal features in metric space.

To investigate the repeatability of the obtained features, an experiment was conducted using 20–25 individual signals from each defect class, assessing and plotting their features in a metric space (Figure 13). The arrangement of these features in the metric space confirmed that all 22 signals generated by the healthy pantograph model were very close to their centroid, which is important because this feature-based system will qualitatively identify a pantograph without defects. Additionally, it should be noted that the features of the first type of defect (a notch caused by a contact wire in the pantograph) were consistently located within a strictly defined area of the feature space from the 23 signals recorded during the experiment, indicating that this type of defect can be reliably identified in the diagnostic system. However, it is essential to point out that the features for defect types 3 and 4 were situated in overlapping spaces, while the arrangement of defect type 2 features partially overlapped with the arrangements for defects 3 and 4.

## 4. Discussion

This means that a study is necessary to reveal the best classifier capable of distinguishing these features. In any case, there is a risk that the implementation of the separation of classes 3 and 4 will have a certain level of error, while the features of defect type 2 will be classified more accurately.

Analyzing the features of defects 2, 3, and 4 in the metric space, it can be inferred that the proposed method will not be able to distinguish them from one another. However, the only common characteristic of these defects is that all of them are critical, involving large deformations, and a pantograph with such defects must be serviced as quickly as possible. Defect 1 could be classified as a “developing defect”: non-critical but the emergence of which could alter the technical service schedule, accelerating the maintenance of the train carrying this pantograph. Therefore, it can be concluded from the experiment that the proposed system can sufficiently accurately differentiate between a healthy pantograph and one with a developing defect, as well as reliably identify a pantograph that requires immediate repair.

## 5. Conclusions

- Separate studies could be conducted to determine the exact values of error probability. However, if the features of defects 2, 3, and 4 are combined into a single aggregate defect class that could be interpreted as “defects requiring technical maintenance”, the proposed method, which evaluates the vibrations generated by the pantograph, would reliably distinguish between a healthy pantograph, a class of single notches, and the “defects requiring technical maintenance” class. Under certain conditions, this may be sufficient for the application task of the technical pantograph diagnostic system.
- The method proposed is limited to the analysis of the signal itself or its spectrum. An alternative approach is suggested for evaluating the characteristics of the vibration signal for diagnostic purposes, and it has been found to be more effective than spectral analysis.

## Data Availability

Dataset available on request from the authors.
